# EIF4A3-Induced Circ_0092278 Enhances Papillary Thyroid Cancer Cell Malignancy by the PI3K/Akt/mTOR Signaling Pathway

**DOI:** 10.4014/jmb.2501.01010

**Published:** 2025-06-19

**Authors:** Mengjiang Liu, Linghui Zhang, Zhaodan Yan

**Affiliations:** Department of Endocrinology, Hubei No. 3 People’s Hospital of Jianghan University, Wuhan 430033, Hubei, P.R. China

**Keywords:** EIF4A3, papillary thyroid cancer, circ_0092278, PI3K/Akt/mTOR

## Abstract

Circular RNA (circRNA) plays a regulatory role in cancer progression, but the role of circ_0092278 in papillary thyroid cancer (PTC) is unclear. In this research, we aimed to reveal the effect of circ_0092278 on PTC progression as well as its interaction with the eukaryotic translation initiation factor 4A3 (EIF4A3). The expression of circ_0092278 in PTC samples was analyzed using quantitative real-time polymerase chain reaction (qRT-PCR). Cell experiments were conducted to assess the effects of circ_0092278 on PTC cell viability, migration, and invasion. Furthermore, key proteins involved in the phosphatidylinositol 3-kinase/protein kinase B/mammalian target of rapamycin (PI3K/AKT/mTOR) pathway were examined using western blotting. Additionally, a xenograft model in nude mice was used to investigate the *in vivo* role of circ_ 0092278. The interactions between EIF4A3 and circ_0092278 were analyzed through RNA-binding protein immunoprecipitation and qRT–PCR assays. Circ_0092278, located in the cell cytoplasm, was significantly upregulated in PTC. Knockdown of circ_0092278 reduced PTC cell proliferation, migration, invasion, and tumor growth by inhibiting the PI3K/Akt/mTOR pathway. Conversely, overexpression of circ_0092278 prompted PTC cell malignancy by activating the PI3K/Akt/mTOR pathway. Additionally, EIF4A3 was found to bind to MINK1 (circ_0092278 linear gene), thereby enhancing the expression of circ_0092278. Circ_0092278, controlled by EIF4A3, promotes PTC progression by activating the PI3K/Akt/mTOR pathway. Our findings indicate that targeting the EIF4A3-circ_0092278 axis may provide a novel approach for treating PTC.

## Introduction

Thyroid cancer (TC) represents 3.4% of all cancers diagnosed each year, with papillary thyroid cancer (PTC) being the most prevalent, accounting for approximately 80% of TC cases [1]. Improvements in medical and surgical practices have allowed researchers to gain a deeper understanding of PTC’s etiology, contributing to a long-term survival rate of over 90% for PTC patients [2, 3]. However, more aggressive mutations in heterogeneous PTC results in a 25% recurrence rate [4, 5]. Thus, further research into the molecular mechanisms driving PTC progression is crucial for improving prognosis and treatment strategies.

Circular RNA (circRNA), a stable and abundant class of noncoding RNAs, plays essential roles in cancer progression by controlling tumor cell behavior [6]. In PTC, the dysregulation of circRNAs—such as circ_0002111, circTIAM1, and circRNA_102002—has been associated with poor clinical outcomes, metastasis, and increased cell malignancy [7-9]. This research identified circ_0092278, also known as hsa_circRNA_400031 and derived from the MINK1 gene, as a key circRNA in PTC through circRNA microarray examination. A review of the literature reviewed only one study that reported the upregulation of circ_0092278 in breast cancer using circRNA microarray analysis [10]. Thus, we investigated the role of circ_0092278 in PTC to expand the regulatory network of this disease’s progression.

Recent research suggests that circRNAs play a role in tumorigenesis by influencing the phosphatidylinositol 3-kinase/protein kinase B/mammalian target of rapamycin (PI3K/AKT/mTOR) pathway [11-13]. The PI3K/AKT/mTOR is a crucial intracellular network that regulates fundamental cellular processes, including, cell proliferation, movement, viability, metallogenesis, and angiogenesis [14]. Studies have indicated that PI3K/AKT/mTOR is activated in PTC and is differentially regulated by various circRNAs, such as circ_100395 [15] and circ_0007694 [16]. However, the role of circ_0092278 in modulating the PI3K/AKT/mTOR pathway in PTC remains to be characterized.

Beyond the signaling pathway, circRNA function is often mediated by RNA-binding proteins [17, 18]. Eukaryotic translation initiation factor 4A3 (EIF4A3) is a key component of RNA shearing and functions as an RNA-binding protein that regulates noncoding RNA levels in tumors [19]. EIF4A3 has been shown to control circASAP1 [20], circARHGAP29 [21], or circ_0087429 [22] at the transcriptional level to influence the progression of glioblastoma, prostate cancer, and cervical cancer. In PTC, only one study has reported that EIF4A3 can induce the expression of has__circ_0118578 to promote tumorigenesis [23]. However, the mechanism of EIF4A3’s effect on circ_0092278 in PTC has yet to be explored.

In this work, we sought to investigate the differential expressions of circ_0092278 in PTC and its regulatory effects on PTC malignancy. Based on bioinformatic analysis and previous research, we hypothesized that circ_0092278 regulated by EIF4A3 may promote PTC malignancy by activating the PI3K/AKT/mTOR pathway. Our findings will support the idea that circ_0092278 can serve as a biomarker for PTC diagnosis.

## Materials and Methods

### Collection of Tissue

A total of 40 PTC patients who underwent surgical treatment at Hubei No. 3 People’s Hospital of Jianghan University were enrolled in this study. All participants provided signed informed consent, and their characteristics are presented in Table 1. This study was approved by the Ethics Committee of the Hubei No. 3 People’s Hospital of Jianghan University (IRB: KY2024007).

### Cell Culture and Transfection

Procell (China) provided two PTC cell lines, TPC-1 (cat. no. CL-0643) and IHH-4 (cat. no. CL-0803). The cells were cultured in RPMI-1640 medium (Procell) supplemented with 10% fetal bovine serum (FBS, Procell) at 37°C in a 5% CO_2_ incubator.

The siRNA-circ_0092278 (si-circ_0092278), a negative control siRNA (si-NC), overexpression vectors for circ_0092278 or EIF4A3 (OE-circ_0092278 or OE-EIF4A3), and an empty vector (used as a negative control of overexpression vector) were synthesized by RiboBio (China). These vectors were transfected into IHH-4 as well as TPC-1 cells (50 nM siRNA or 2 μg/ml overexpression vector) using the Lipo6000 reagent (Beyotime, China). At 48 h post-transfection, the treated cells were collected for subsequent assays.

### Quantitative Real-Time Polymerase Chain Reaction (qRT-PCR) for Gene Expression Detection

Following the manufacturer’s protocol, total RNA was extracted from cells and tissues using an RNA extraction kit (CWBIO, China). RNAs were then reverse-transcribed using the PrimeScript 1^st^ Strand cDNA Synthesis Kit (Takara, Japan). Following that, qRT–PCR was performed using SYBR Green PCR Master Mix (Qiagen, USA). Gene expression levels were calculated using the 2^-ΔΔCt^ method with GAPDH used for normalization. The primers are listed in Table 2.

### RNase R Treatment to Identify the Stability of Circ_0092278

Cellular RNA was treated with RNase R from Beyotime or PBS (for the MOCK group), and RNA digestion was carried out at 37°C for 30 min to eliminate linear RNA. Afterward, the remaining RNA was amplified to generate cDNA. The levels of circ_0092278 and its linear transcript (MINK1) were then measured using qRT-PCR.

### Nuclear–Cytoplasmic Fractionation to Detect the Location of Circ_0092278

Nuclear cytoplasmic separation was performed to determine the location of circ_0092278 in PTC cells. The PARIS Kit (Invitrogen, USA) was used to isolate the cytoplasm as well as the nucleus of PTC cells. Briefly, 1 × 10^6^ TPC-1 as well as IHH-4 cells were incubated with 500 μl of cell disruption buffer for 10 min. After centrifugation (500 ×g) at 4°C, the cytoplasmic fraction was collected from the supernatant, and the nuclear fraction was collected from the pellet. To confirm the efficacy of the separation, GAPDH was used as a marker for the cytoplasm, and U6 served as a marker for the nucleus. The purity of the fractions was validated by assessing the enrichment of GAPDH in the cytoplasmic fraction and U6 in the nuclear fraction using qRT–PCR. The relative expressions of circ_0092278 were then quantified in each fraction.

### Cell Counting Kit-8 (CCK-8) Assay for PTC Cell Proliferation Detection

A CCK-8 kit (Beyotome) was used to assess the proliferation of PTC cells. IHH-4 and TPC-1 cells were plated into 96-well plates (5,000 cells/well). The cells were cultured for 0 h, 24 h, 48 h, 72 h, and 96 h. Following removal of the cultured medium, a 100 μl RPMI-1640 medium containing 10 μl CCK-8 solution was added. After an additional 90 min incubation, a microplate reader (Bio-Rad Laboratories, USA) was used to measure the absorbance at 450 nm.

### Wound-Healing Assay for PTC Cell Migration Detection

TPC-1 and IHH-4 cells were seeded into a 6-well plate at a density of 2 × 10^4^ cells/well and cultured for 48 h to allow monolayer formation. Scratches were then made using a 10-μl tip. The wound areas were photographed at 0 h and 24 h post-scratch using a light microscope (Olympus, Japan). The wound closure rate was subsequently calculated.

### Transwell Assay for PTC Cell Invasion Detection

IHH-4 and TPC-1 cells were incubated in a serum-free basal medium for 6-8 h to eliminate residual serum. The cells were then resuspended in the basal medium at a concentration of 1 × 10^5^/ml. An aliquot (200 μl) of the cell suspension was added to the chamber of a transwell insert pre-coated with Matrigel. The insert was then placed into the lower chamber containing 500 μl medium supplemented with 20% FBS. After 24 h incubation, the invading cells were fixed with 4% paraformaldehyde for 15 min and stained with 1% crystal violet for 30 min. Finally, the number of invading cells was counted using a light microscope (Olympus).

### Western Blot Analysis of Protein Expression

The protein concentrations of C cells and tumor tissues were assessed using the BCA assay after cell lysis with RIPA buffer (Beyotime). An equal amount of protein (50 μg) was separated on 12% SDS-PAGE and transferred onto PVDF membranes, which were then blocked for 1 h in a solution containing 5% BSA and 0.1% Tween-20. Next, the membranes were incubated overnight at 4°C with the following primary antibodies from Beyotime: Phospho-PI3K (AF5905), PI3K (AF7742), AKT (AA326), Phospho-AKT (AA329), mTOR (AF1648), Phospho-mTOR (AF5869), and GAPDH (AF1186). Following that, the membranes were treated with a rabbit secondary antibody (Beyotime) at 25°C for 2 h. The protein bands were visualized using an ECL detection system (Pierce Biotech, USA), and their intensities were quantified by densitometric analysis with the Gel-Pro analyzer (version 4.0, Gel Media Cybernetics, USA).

### Animal Experiments for Tumor Growth Detection *In Vivo*

Animal experiments involving nude mice were approved by the Animal Care and Use Committee of Hubei No. 3 People’s Hospital of Jianghan University (IRB: 202406). The shRNA targeting circ_0092278 (sh-circ_0092278, identical to the si-circ_0092278 target) and the negative control (sh-NC) were inserted into the pLL3.7 lentivirus vector. In addition, the circ_0092278 overexpression sequence was inserted into the pBOBI lentiviral vector (LV-circ_0092278), with an empty vector used as the control (LV-NC). IHH-4 cell lines expressing sh-circ_0092278, LV-circ_0092278, sh-NC, or LV-NC were then established. Twelve male BALB/c nude mice (~20 g each) obtained from Hunan SJA Laboratory Animal Center (China), were subcutaneously injected with IHH-4 cells (1.0×10^7^cells/150 μl PBS). Tumor growth was assessed weekly. After five weeks, the mice were euthanized, and tumor volume and weight were measured.

### RNA-Binding Protein Immunoprecipitation (RIP) Assay to Detect the Interaction of circ_0092278 and EIF4A3

The interaction between circ_0092278 and EIF4A3 was examined using the Magna RIP Kit (Shanghai Advantage Biotech., China). In brief, cells were lysed with RIP lysis buffer, and the resulting lysates were incubated overnight at 4°C with magnetic beads bound to either anti-EIF4A3 antibody (abs155050, Absin) or IgG (ab134050, Abcam). Following incubation, RNA was extracted from the immunoprecipitated complexes for subsequent quantification of circ_0092278 expression.

### Statistical Analysis

All data (expressed as mean ± SD) from the experiment were performed in triplicate. Statistical analysis was conducted using SPSS software (IBM, USA). Comparisons between multiple groups were evaluated using one-way ANOVA followed by Tukey’s post-hoc test, while comparisons between two groups were assessed using Student’s *t*-test. A *p*-value of <0.05 was considered significant.

## Results

### Circ_0092278 Is Upregulated in PTC

**Fig. 1:**
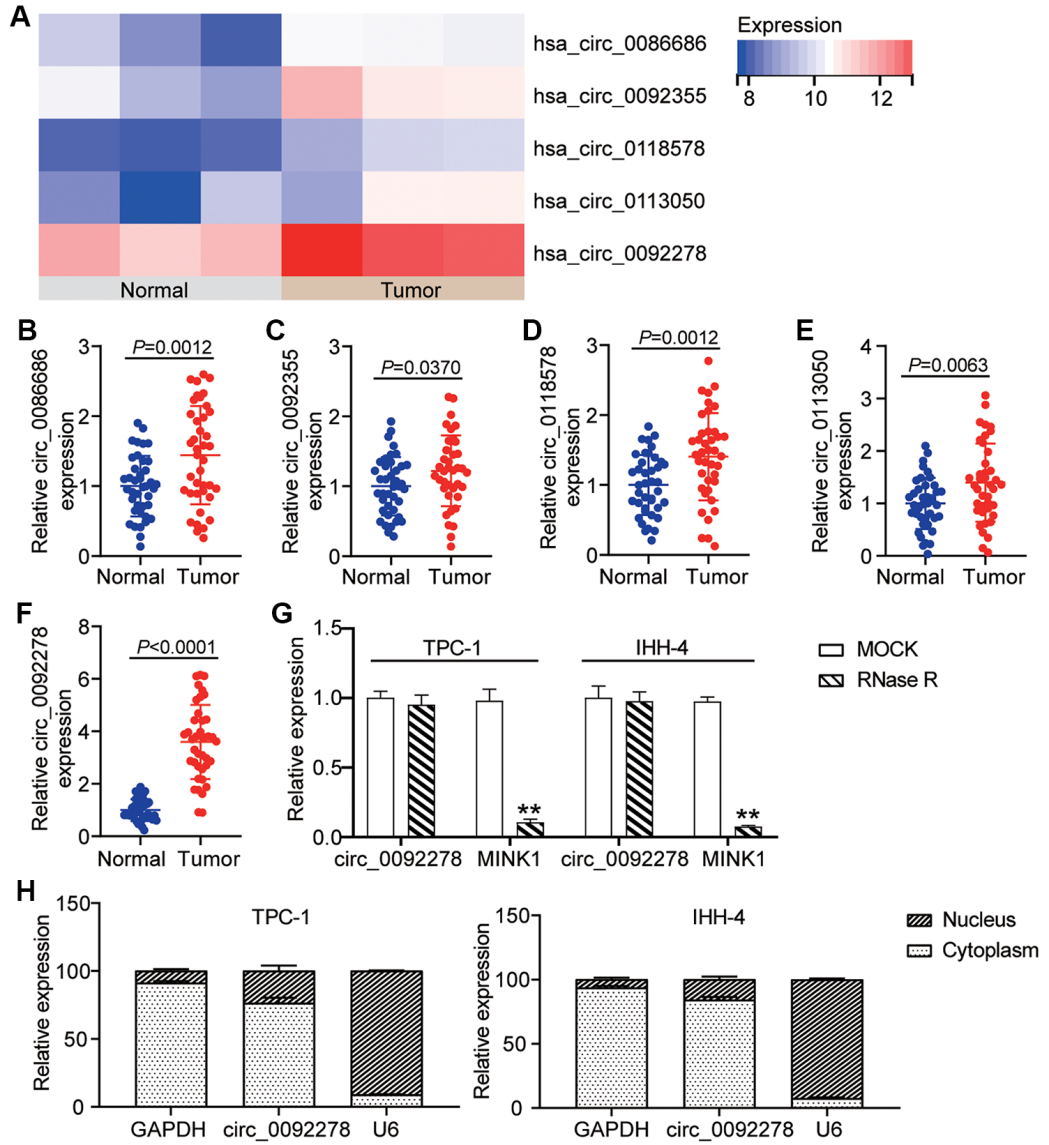
Circ_0092278 is upregulated in PTC. (**A**) The top 5 upregulated circRNAs in PTC samples were confirmed according to the data from GSE173299. (**B-F**) circ_0086686 (**B**) circ_0092355 (**C**) circ_01118578 (**D**) circ_0223050 (**E**) circ_0092278 (**F**) levels in PTC tissue and normal tissue samples were revealed via qRT-PCR. (**G**) Circ_0092278 and MINK1 (linear transcript of circ_0092278) levels in IHH-4 and TPC-1cells after RNase R treatment. **p < 0.001 vs. MOCK. (**H**) Nucleocytoplasmic localization analysis of circ_0092278 in IHH-4 and TPC-1cells.

According to the data from GSE173299, the five most significantly upregulated circRNAs in the PTC samples were confirmed (Fig. 1A). Subsequent qRT-PCR analysis revealed that circ_0086686, circ_0092355, circ_01118578, circ_0223050, and circ_0092278 were upregulated by 1.4-, 1.8-, 2.0-, 2.1- and 5.0-fold, respectively, in the PTC tissues (Fig. 1B-1F). Among these, circ_0092278 showed the highest expression and was suggested for further investigation. Correlation analysis revealed that elevated circ_0092278 expression was significantly associated with larger tumor size, lymphovascular invasion, and lymph node metastasis (Table 3). Furthermore, RNase R treatment demonstrated that circ_0092278 was resistant to degradation, unlike its linear counterpart, MINK1 (Fig. 1G). As depicted in Fig. 1H, circ_0092278 was predominantly localized in the cytoplasm of both TPC-1 and IHH-4 cells. Collectively, these findings suggest that elevated circ_0092278 may contribute to the malignant progression of PTC.

### Circ_0092278 Upregulation Attenuates PTC Cell Malignancy, While Silencing Accelerates It

**Fig. 2:**
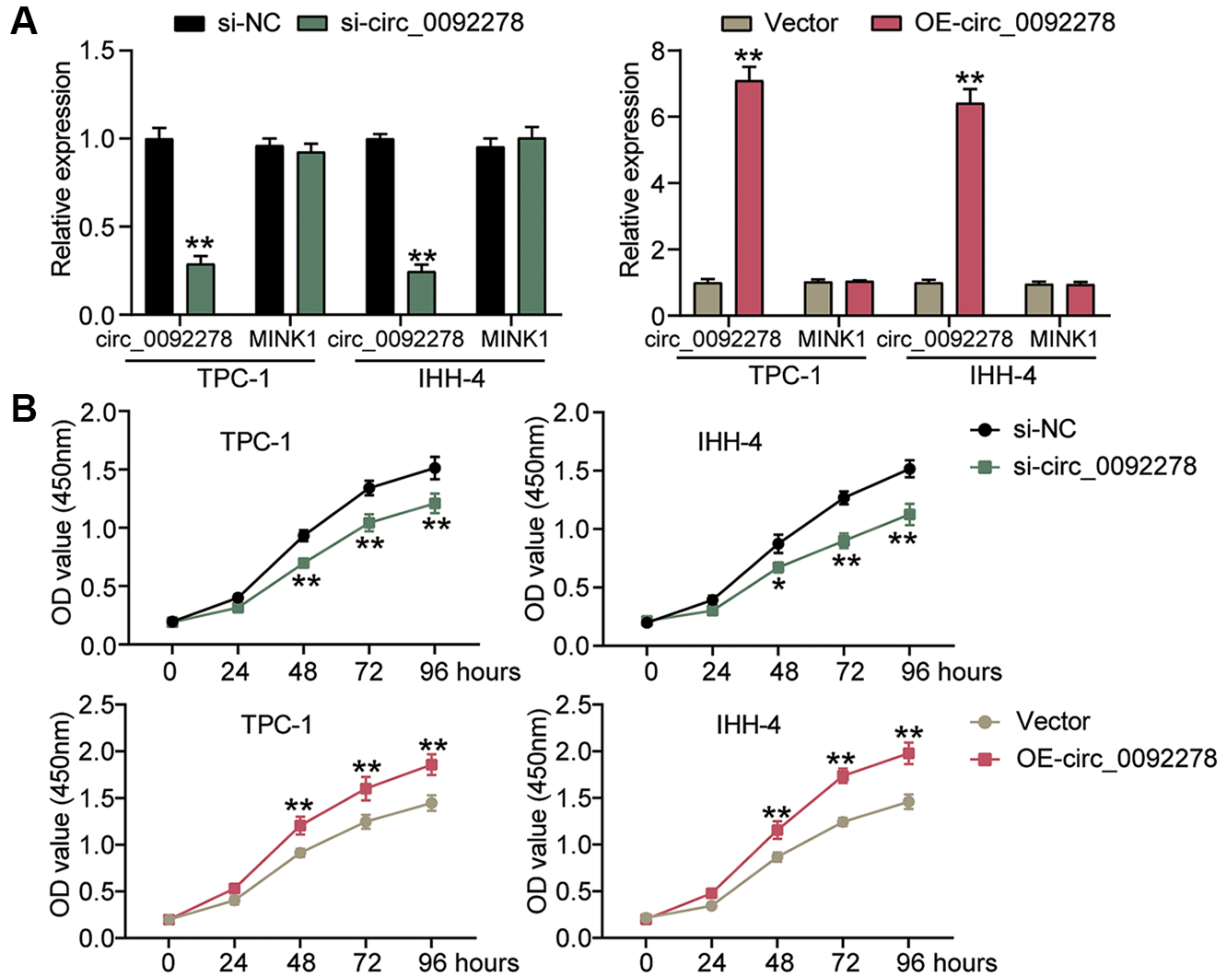
Circ_0092278 upregulating attenuates GC cell proliferation, while silencing accelerates it. (**A**) The levels of circ_0092278 in IHH-4 and TPC-1 cells delivered with si-circ_0092278 or OE-circ_0092278 were determined via qRTPCR. (**B**) Cell proliferation in IHH-4 with TPC-1 cells delivered with si-circ_0092278 or OE-circ_0092278 was confirmed using CCK-8 assays. **p < 0.001 vs. si-NC/Vector.

**Fig. 3:**
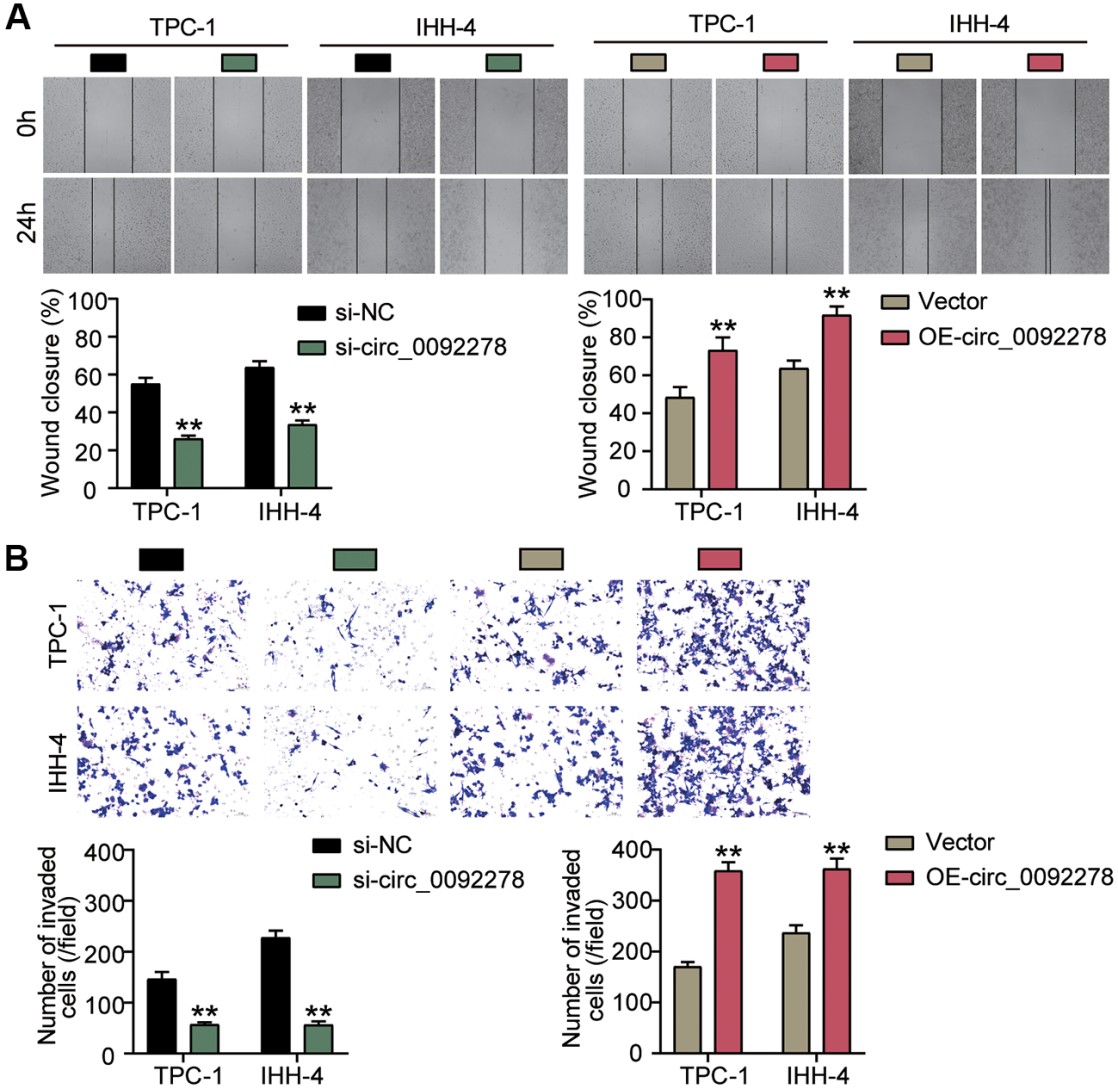
Circ_0092278 upregulating attenuates GC cell migration and invasion, while silencing accelerates it. (**A**) Cell migration in IHH-4 and TPC-1cells delivered with si-circ_0092278 or OE-circ_0092278 was assessed through wound healing assays. (**B**) Cell invasion in IHH-4 and TPC-1cells delivered with si-circ_0092278 or OE-circ_0092278 was confirmed by Transwell assays. **p < 0.001 vs. si-NC/Vector.

In light of the abnormal expression of circ_0092278 in PTC, we conducted loss-of-function and gain-of-function assays to investigate its impact on the malignant characteristics of PTC cells. To modulate circ_0092278 levels, PTC cells were transfected with si-circ_0092278, OE-circ_0092278, si-NC, or OE-NC into the tumor cells. As shown in Fig. 2A, si-circ_0092278 effectively reduced circ_0092278 expression by >60%, while OE-circ_0092278 increased its expression by approximately 7-fold. Functional assays demonstrated that silencing circ_0092278 led to a reduction in PTC cell proliferation by >20%, whereas its overexpression boosted proliferation by >1.3-fold (Fig. 2B). Moreover, the knockdown of circ_0092278 reduced the migration ability of IHH-4 and TPC-1 cells by nearly 50%, and their invasive capacity by >60% (Fig. 3A and 3B). Conversely, overexpression of circ_0092278 enhanced cell migration by >1.3-fold, and increased invasiveness by >1.5-fold (Fig. 3A and 3B). Overall, these findings indicate that circ_0092278 knockdown inhibits, while its overexpression promotes, the malignant behavior of PTC cells *in vitro*.

### Circ_0092278 Upregulation Inactivates the PI3K/AKT/mTOR Pathway in PTC Cells, but Silencing Motivates It

**Fig. 4:**
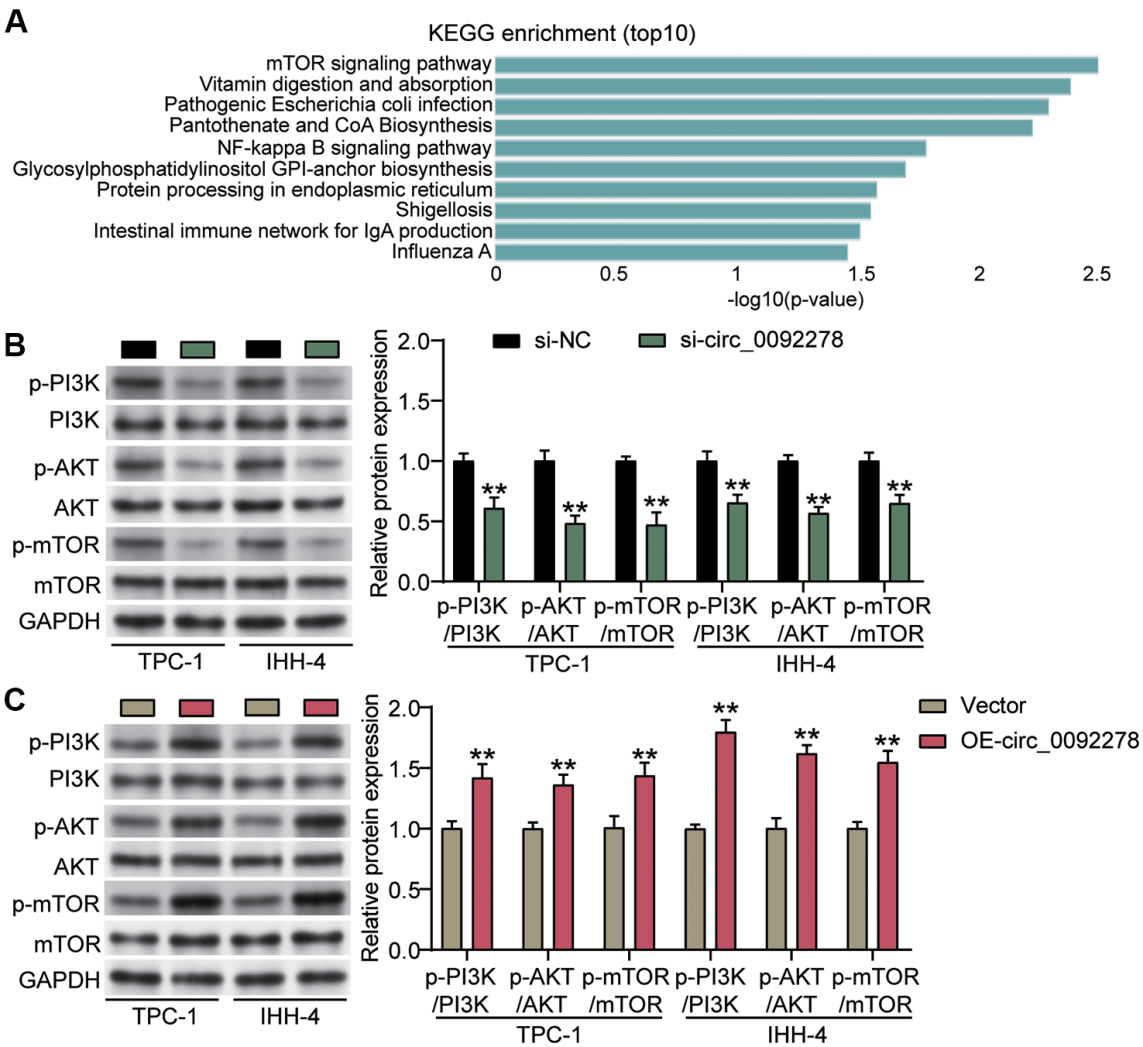
Circ_0092278 upregulating inactivates the PI3K/AKT/mTOR pathway in PTC cells while silencing motivates it. (**A**) CircInteractome predicted potential miRNAs target of circ_0092278, while KEGG enrichment analysis of these miRNAs was conducted using miEAA. (**B**) Western blotting analysis of p-PI3K, p-AKT, p-mTOR, PI3K, AKT, and mTOR expression in IHH-4 and TPC-1 cells delivered with OE-circ_0092278. **p < 0.001 vs. si-NC. (**C**) Western blotting analysis of p- PI3K, p-AKT, p-mTOR, PI3K, AKT, and mTOR expression in IHH-4 and TPC-1 cells delivered with si-circ_0092278. **p < 0.001 vs. Vector.

Potential miRNAs interacting with circ_0092278 were identified using circInteractome (https://circinteractome.nia.nih.gov/index.html). Subsequent KEGG pathway enrichment analysis of potential miRNAs, performed using miEAA (https://ccb-compute2.cs.uni-saarland.de/mieaa/downloads/), highlighted the mTOR signaling pathway as the most enriched (Fig. 4A). Given that mTOR serves as a crucial downstream effector of the PI3K/Akt/mTOR pathway, this enrichment pointed toward circ_0092278 having a regulatory involvement in this pathway. Western blot analysis further confirmed that circ_0092278 led to decreased phosphorylation levels of P13K, AKT, and mTOR, whereas its overexpression resulted in increased phosphorylation of these proteins (Fig. 4B and 4C). Additionally, clinical data from PTC patients showed a positive association between high circ_0092278 expression and elevated phosphorylation of PI3K, AKT, and mTOR (Table 3). These results indicate that the downregulation of circ_0092278 inactivates the induced PI3K/AKT/mTOR signaling pathway in PTC cells, while its upregulation has an activating effect.

### Circ_0092278 Overexpression Reduces Tumor Growth, Whereas Knockdown Enhances It

**Fig. 5:**
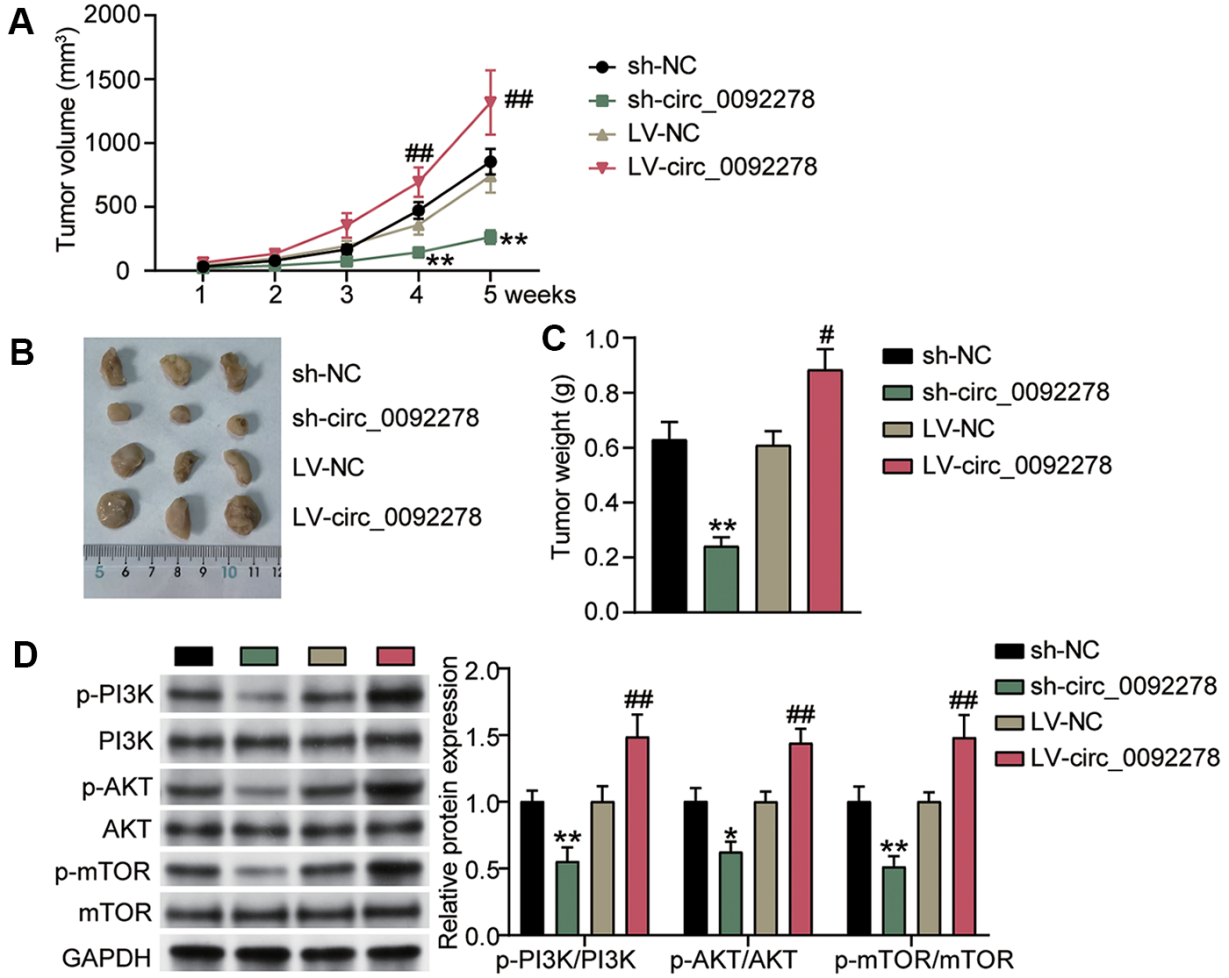
Circ_0092278 overexpression reduces tumor growth, while knockdown enhances it. (**A**) Tumor growth in the sh-circ_0092278 and LV-circ_0092278 groups was measured every week. (**B**) Images of xenograft tumors in mice that were subcutaneously injected with sh-circ_0092278 or LV-circ_0092278 group IHH-4 cells. (**C**) After maintaining mice for 5 weeks, the tumoral weight at the time of euthanasia was measured in the sh-circ_0092278 or LV-circ_0092278 groups. (**D**) Western blotting analysis of p-PI3K, p-AKT, p-mTOR, PI3K, AKT, mTOR expression in the sh-circ_0092278 or LVcirc_ 0092278 group mice. *p < 0.05 vs. sh-NC, **p < 0.001 vs. sh-NC; #*p* < 0.05 vs. LV-NC, ##*p* < 0.001 vs. LV-NC.

Furthermore, we tested the oncogenic potential of circ_0092278 *in vivo* and subcutaneously injected sh-circ_0092278, LV-circ_0092278, sh-NC, or LV-NC IHH-4 cells into mice. Indeed, stable knockdown of circ_0092278 decreased tumor volume (Fig. 5A), size (Fig. 5B), and weight (Fig. 5C). Meanwhile, overexpression of circ_0092278 increased tumor volume, size, and weight. Furthermore, western blotting revealed that p-mTOR, p-AKT, and p-PI3K levels in tumor tissues were all reduced or increased following circ_0092278 silencing or overexpression (Fig. 5D). That is, circ_0092278 knockdown inhibited PTC cell growth by activating the PI3K/AKT/mTOR network.

### EIF4A3 Binds to MINK1 (circ_0092278 Linear Gene)

**Fig. 6:**
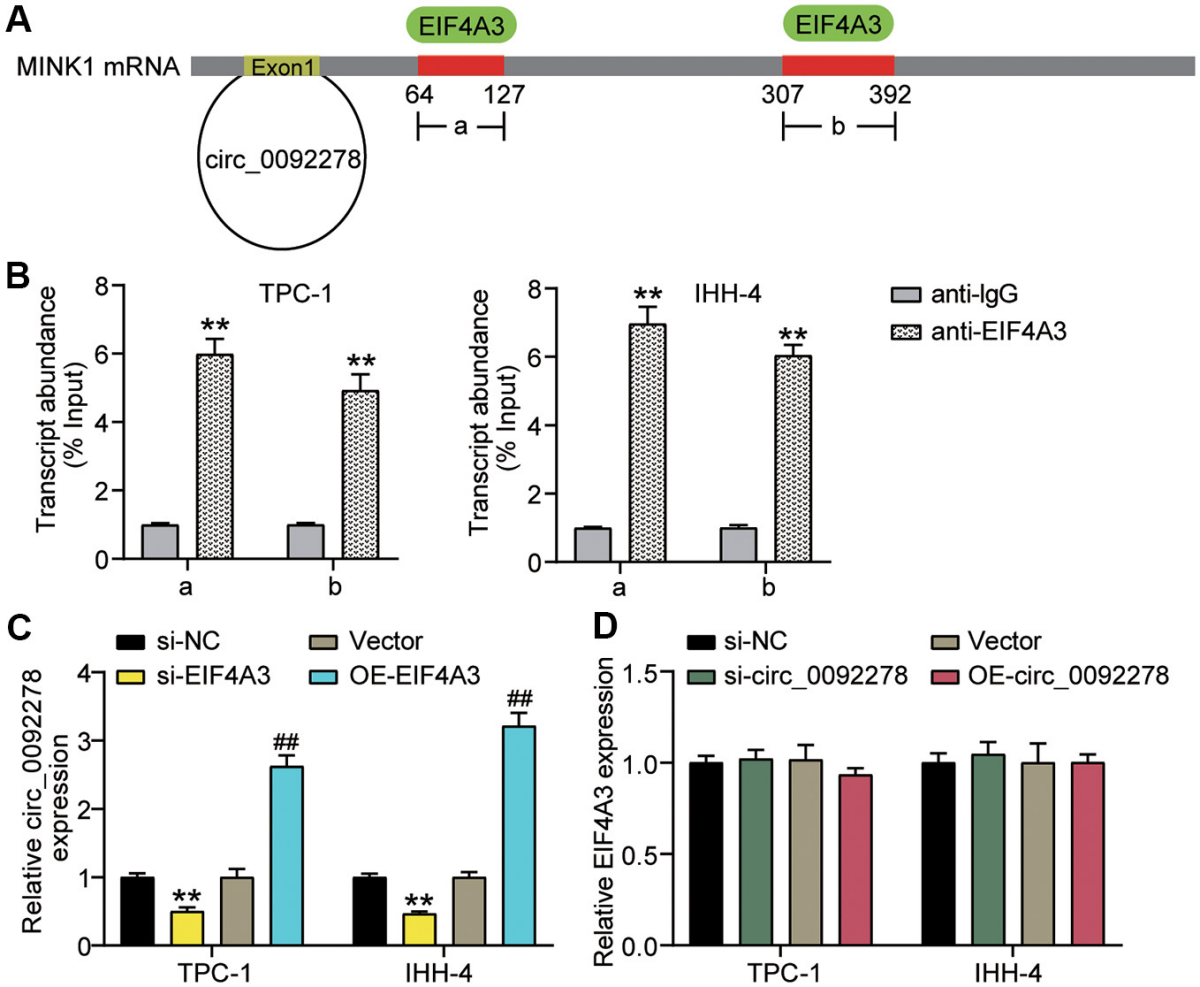
EIF4A3 binds to MINK1 (circ_0092278 linear gene). (**A**) circInteractome predicts that EIF4A3 could bind to the flanking sequences of MINK1 mRNA (circ_0092278 linear gene). (**B**) RIP assays showing the interaction between MINK1 mRNA (circ_0092278 linear gene) binds EIF4A3 in IHH-4 and TPC-1 cells. **p < 0.001 vs. anti-IgG. (**C**) Circ_0092278 levels in IHH-4 and TPC-1 cells delivered with si-EIF4A3 or OE-EIF4A3 were revealed via qRT-PCR. **p < 0.001 vs. si-NC/Vector. (**D**) EIF4A3 levels in IHH-4 and TPC-1 cells delivered with si-circ_0092278 or OE-circ_0092278 were analyzed via qRT–PCR.

circInteractome predicted that EIF4A3 could bind to the flanking sequences of MINK1 mRNA (circ_0092278 linear gene) and has two binding sites (site a, base sites 64 to 127; site b, base sites 307 to 392) (Fig. 6A). RIP was used to assess the binding of MINK1 mRNA to EIF4A3, and the transcript abundance of MINK1 mRNA with anti-EIF4A3 was increased approximately 6- and 5-fold, respectively, in sites a and b (Fig. 6B). Furthermore, the regulatory relationship was determined using qRT-PCR, and the data revealed that circ_0092278 expression was reduced when EIF4A3 was silenced, and increased when EIF4A3 was overexpressed (Fig. 6C). In comparison, the EIF4A3 levels remained stable when circ_0092278 was silent or highly active (Fig. 6D). In conclusion, EIF4A3 targets MINK1 mRNA (circ_0092278 linear gene) and increases its levels in PTC cells.

## Discussion

To look for new predictive biomarkers and better understand the complex molecular mechanisms underlying PTC progression, we investigated a newly discovered circRNA, circ_0092278, which had never been characterized in PTC. Circ_0092278 was found to be overexpressed in PTC tissues, contributing to tumor cell proliferation, migration, invasion, and growth. Furthermore, bioinformatics analysis revealed that circ_0092278 regulates the PI3K/Akt/mTOR network, and western blotting was used to confirm that a level of circ_0092278 activates it. Additionally, EIF4A3 may increase the level of circ_0092278 by binding to the MINK1 (circ_0092278 linear gene) sequence. Collectively, our findings contribute to a fuller understanding of PTC progression and may suggest a therapeutic target for PTC patients.

CircRNAs are extensively studied as critical components of cancer-associated bio-processes resulting from reverse splicing [24]. Furthermore, the ability to characterize and structurally stabilize abnormal levels in multiple tumors provides significant advantages for circRNAs as cancer targets [25]. There is strong evidence that circRNAs interfere with the onset and progression of PTC. Wen *et al*. [26] found that suppressing overexpressed circRNA_102171 in PTC increased apoptosis while inhibiting tumor cell tumorigenicity, survival, and metastasis. Overexpression also facilitated PTC malignancy by activating the Wnt/β-catenin network. Meanwhile, data from Yao *et al*. [27] revealed that hsa_circ_0011385 interacts with AUF1, thereby promoting PTC cell cycle progression, proliferation, migration, and invasion *in vitro*. Chu *et al*. [28] showed that circPCNXL2 meets the energy metabolism requirements of PTC cells to promote tumor cell growth. We first discovered that circ_0092278 was highly regulated in PTC samples, and the functional experiments revealed that circ_0092278 defects significantly reduced invasion, proliferation, and metastasis, as well as cancer cell survival *in vivo*. Furthermore, circ_0092278 upregulation accelerated the progression of malignant PTC cells, thereby promoting tumor growth.

Hyperactivation of the PI3K/AKT/mTOR network occurs in almost all malignant tumors and is essential for cell motility, growth, survival, and metabolism in patients with cancer [29, 30]. Research has shown that the modified PI3K/AKT/mTOR network is facilitated in PTC, and pathway inhibition reduces the malignant behavior of PTC cells [31, 32]. Furthermore, circRNA influences PI3K/AKT/mTOR regulation of PTC cell function. Based on KEGG analysis and protein blotting, Long *et al*. [16] confirmed circ_0007694 as inhibiting the PI3K/AKT/mTOR network to prevent PTC development. Meanwhile, Chen *et al*. [15] revealed that the PI3K/AKT/mTOR network was dampened and alleviated aerobic glycolysis in PTC cells following the upregulation of circ_100395. In this research, KEGG analysis showed that the PI3K/AKT/mTOR network was regulated by circ_0092278. Western blot results further demonstrated that increased circ_0092278 expression elevated the phosphorylation of the key proteins (mTOR, AKT, PI3K) of the PI3K/AKT/mTOR pathway while silencing circ_0092278 suppressed their phosphorylation. Consistently, PTC cells with altered circ_0092278 expression led to corresponding changes in the levels of phosphorylated PI3K/AKT/mTOR in tumor tissues following *in vivo* tumor formation. These findings suggest that circ_0092278 may promote PTC progression by activating the PI3K/AKT/mTOR signaling cascade.

Abnormal mRNA translation is a major contributor to tumor malignancy, with alterations in EIF4A family members often leading to excessive oncogenic mRNA activity [33]. EIF4A3, a crucial component of the exon junction complex (EJC), plays a vital role in circRNA formation by binding to precursor and mRNAs and promoting back-splicing events [34, 35]. For instance, Jiang *et al*. [36] reported that EIF4A3 facilitated the generation of circ_0084615 in colorectal cancer by binding to its host gene mRNA ASPH, thereby contributing to tumor development. In the context of PTC, only Li *et al*. [23] showed that EIF4A3 stabilizes circ_0118578 by interacting with its precursor mRNA SATB2, thereby promoting tumor growth. Similarly, our study discovered that EIF4A3 also interacts with circ_0092278, playing a role in PTC progression. Specifically, EIF4A3 binds to the MINK1 transcript (the host mRNA of circ_0092278) during splicing, aiding in the identification of back-splice sites and supporting circ_0092278 formation. Additionally, EIF4A3 stabilized circ_0092278 after its synthesis, thereby increasing its expansion. These findings are consistent with established models of EIF4A3-mediated circRNA biogenesis, in which EJC assembly at the splice site promotes circRNA production.

While this research sheds light on the mechanistic role of circ_0092278 in PTC, several limitations should be acknowledged and addressed in future research. First, the lack of validation in primary patient samples restricts the clinical relevance of our findings. Although cell lines and xenograft models provide valuable mechanistic insights, they may not fully capture the complexity of PTC’s heterogeneity and microenvironment. Further studies should focus on correlating circ_0092278 expression with patient survival, histopathological subtypes (e.g., aggressive variants), and treatment responses in clinical PTC samples. To advance the clinical translation of circ_0092278, further research should assess its potential as a biomarker for risk stratification or therapeutic response monitoring. Additionally, given emerging evidence that certain drugs such as spleen tyrosine kinase [37] and aryl hydrocarbon receptor antagonists [38] can regulate circRNAs in PTC, future investigation should examine whether pharmacologic agents influence circ_0092278 expression and whether such modulation enhances existing therapies. Finally, future studies will need to investigate the feasibility of directly targeting circ_0092278 using antisense oligonucleotides to evaluate its potential as a therapeutic target in preclinical models.

In summary, our research demonstrated that circ_0092278 is upregulated in PTC, and its overexpression promotes tumor cell proliferation, invasion, and migration by activating the PI3K/AKT/mTOR pathway. Furthermore, EIF4A3 serves as an upstream positive regulator of circ_0092278. This study identifies a potential mechanism of PTC progression through the EIF4A3-circ_0092278-PI3K/AKT/mTOR axis and suggests a promising molecular target for PTC diagnosis and treatment.

## Figures and Tables

**Table 1:** Characteristics of papillary thyroid cancer individuals (*n* = 40).

Variable	N (%)
Age (years, mean ± SD)	47.8 ± 11.2
Sex	
Male	6 (15.0)
Female	34 (85.0)
Tumor size (mm)	
<1	24 (60.0)
≥1	16 (40.0)
Multifocality	
Single	22 (55.0)
Multiple	18 (45.0)
Lymphovascular invasion	
Yes	7 (17.5)
No	33 (83.5)
Lymph node metastasis	
Yes	17 (42.5)
No	23 (57.5)

**Table 2:** Primers for qRT-PCR.

Gene	Primer	Sequence (5'-3')
circ_0086686	Forward	CACGGCTACAGTACAGGTCC
	Reverse	AGGTTCACCCCATGTTGCTT
circ_0092355	Forward	AGTTTGCCACGATATTGATGTG
	Reverse	AAAGAAAGGAGTGGAGGGCA
circ_0118578	Forward	TGGAACCATGCCACAGTCC
	Reverse	CACATCTTTCCGCACCAGG
circ_0113050	Forward	AGAGGAGTGACAAAGCTGGA
	Reverse	GAATTGGTCTTGAGAAGGGCC
circ_0092278	Forward	CTGCGTATTATGAGGTGCCA
	Reverse	GGCTATGCTTTGTGAGGCTG
MINK1	Forward	CTGATGTTGCTGGACCGAAG
	Reverse	AGAATCTTGTTCCGGAGCCA
EIF4A3	Forward	CCGCATCTTGGTGAAACGTGAT
	Reverse	GCCTGAGTGATGGTCAGTGTGT
GAPDH	Forward	AAATCAAGTGGGGCGATGCTG
	Reverse	GCAGGAGGCATTGCTGATGAT
U6	Forward	GGAACGATACAGAGAAGATTAGC
	Reverse	TGGAACGCTTCACGAATTTGCG

**Table 3:** The correlation between circ_0092278 expression and pathological features.

Variable	circ_0092278 expression		*p*-value
	High	Low	
Age			0.5231
<47.8	10	7	
≥47.8	10	13	
Sex			0.6614
Male	4	2	
Female	16	18	
Tumor size (mm)			0.0031
<1	7	17	
≥1	13	3	
Lymphovascular invasion			0.0083
Yes	7	0	
No	13	20	
Lymph node metastasis			0.0011
Yes	14	3	
No	6	17	
Phosphorylation of PI3K			0.0033
Yes	20	12	
No	0	8	
Phosphorylation of AKT			0.0197
Yes	19	12	
No	1	8	
Phosphorylation of mTOR			0.0436
Yes	19	13	
No	1	7	
